# Gene promoters are genomically encoded to facilitate histone exchange/incorporation

**DOI:** 10.1371/journal.pbio.3003160

**Published:** 2025-05-13

**Authors:** Blaine Bartholomew

**Affiliations:** University of Texas, MD Anderson Cancer Center, Houston, Texas, United States of America

## Abstract

Gene promoter regions are intrinsically hardwired not only to facilitate transcription preinitiation complex formation, but also the release of paused RNA polymerase by H2A.Z. Ed Luk and colleagues reveal poly (dA) tracks at promoters positively stimulates the incorporation of H2A.Z by the SWR complex in Saccharomyces cerevisiae.

## Teaser

Gene promoters are intrinsically hardwired not only to facilitate transcription preinitiation complex formation but also the release of paused RNA polymerase by H2A.Z. A new PLOS Biology study reveals poly (dA) tracts at promoters positively stimulate H2A.Z incorporation by the SWR complex in yeast.

Genomic DNA is package into chromatin by nucleosomes in which DNA is wrapped ~1.7 times around a protein disk comprised of eight histone proteins. As early as 2006, there was evidence for genomic positioning of nucleosomes to be intrinsically determined at the DNA sequence level [1]. Nucleosome positioning in some cases is linked to the ability of certain DNA sequences to naturally bend and adopt the conformation necessary for efficiently binding to the histone octamers or to preclude binding of the histone octamer [2]. There are, however, many instances where DNA sequence alone is insufficient to explain the genomic chromatin landscape. For example, one study found that reconstituting chromatin with DNA and recombinant histone octamers is alone not sufficient to accurately reconstitute the in vivo state of chromatin [3]. ATP-dependent chromatin remodelers are the missing factor, and when added with ATP to in vitro assembled yeast chromatin produces nearly the same pattern as observed in vivo.

ATP-dependent remodelers only mobilize nucleosomes (ISWI, CHD1, and INO80) or mobilize and displace nucleosomes (SWI/SNF) or exchange out specific histones from nucleosomes (SWR in yeast and p400 and SRCAP in vertebrates, and INO80). A common DNA motif found at gene promoters in *Saccharomyces cerevisiae* is a poly d(A) tract, which has been shown in vitro to halt nucleosome movement by the RSC complex [3], a member of the SWI/SNF family, and Chd1 [4]. A poly (dA) tract and a GC-rich motif at promoter regions were also shown in vivo to regulate nucleosome positioning by SWI/SNF [5]. INO80 is another chromatin remodeler that halts nucleosome movement in the promoter region and accurately positions nucleosomes at the same +1 position as observed in vivo [3]. This property of INO80, however, is not mediated by INO80’s affinity for a consensus DNA sequence but is instead dictated by the physical bending properties of DNA [6]. These data show the DNA at gene promoters directly modulates the different chromatin remodelers that act at this region.

**Fig 1:**
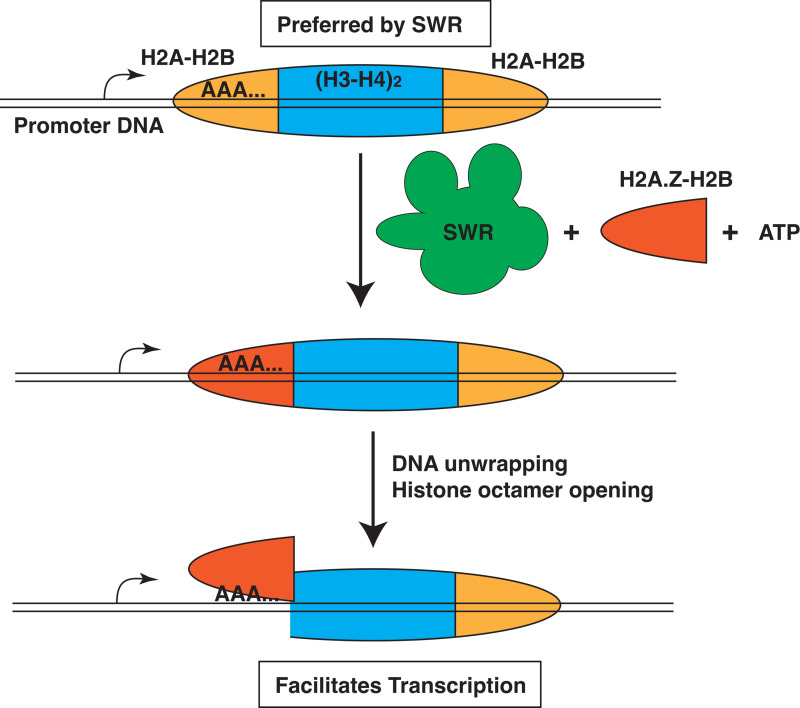
The DNA sequence preference of SWR chromatin remodeler at promoters. The SWR complex prefers to remove H2A-H2B dimers (orange) at the side of nucleosomes where there is a poly (dA:dT) tract (AAA…). SWR complex replaces the H2A-H2B dimer with an H2A.Z-H2B dimer (red). The incorporation of H2A.Z-H2B dimer at the side of the nucleosome closest to the start site of transcription causes that side of the nucleosome to be more prone for DNA to unwrap from the histone octamer and for the histone octamer to form a more open conformation. These changes in the nucleosome structure facilitates in transcription initiation.

SWR also functions at promoter regions, but rather than mobilizing nucleosomes, removes H2A and replaces it with H2A.Z in an ATP-dependent manner [7]. Histones are often classified as either canonical (expressed and incorporated during the S-phase of the cell cycle) or as a variant (constitutively expressed and incorporated outside of the S-phase) [8]. H2A.Z is an H2A variant that shares 60% identity with H2A and has major roles in transcription, heterochromatin boundaries, chromosome segregation, and double-stranded break repair [9]. Incorporation of H2A.Z into nucleosomes increases DNA unwrapping from the histone octamer, which makes DNA more accessible to the transcriptional machinery (see Fig 1) [10]. In H2A.Z nucleosomes, histone H2A.Z-HB dimers have weakened interactions with the H3-H4 tetramer and a higher tendency to pivot away from the tetramer that also leads to increased DNA accessibility [10].

The incorporation of H2A.Z was previously found to prefer the side of nucleosomes where there is a run of >3 consecutive G:C spanning across a 16-base pair region [11]. It was not clear if this DNA sequence specificity reflected that observed in vivo because it was selectively seen at lower temperatures and used Widom’s artificial nucleosome positioning 601 sequence [11]. Now, using a combination of in vivo and in vitro assays, a new PLOS Biology study shows that poly (dA:dT) stimulates H2A.Z incorporation into native yeast mononucleosomes [12]. The authors developed a novel method to map the extent of one or two copies of H2A.Z being incorporated per nucleosome in vivo. They engineered unique cysteines into residues 46 of H2A.Z and 39 of H2A to selectively disulfide crosslink the two copies of H2A present in an individual nucleosome. H2A.Z is also distinguished from H2A by fusing two copies of the V5 epitope to the C-terminus of H2A.Z. Here, the authors find that most H2A.Z nucleosomes contain only one copy of H2A.Z, which is ~5 times more likely to occur than nucleosomes with all H2A replaced with H2A.Z. As well as showing (by MNase ChIP-seq) that SWR is responsible for incorporating H2A.Z at the +1 position of promoters, as observed previously, they also identify clusters of new sites not previously reported to be SWR-dependent that are not linked to promoter regions or histone acetylation, which they refer to as H2A.Z islands.

The sequence bias of SWR was delineated using native H2A mono-nucleosomes isolated by micrococcal nuclease digestion of native yeast chromatin from cells that had both Swr1, the catalytic subunit of the SWR complex, and histone H2A.Z removed. Here, the authors find that SWR prefers incorporating H2A.Z into nucleosomes containing poly (dA:dT) tracts proximal to the DNA entry and exit sides (Fig 1). They perform additional experiments to examine the potential of poly (dA:dT) to regulate SWR using 601 nucleosomes and introduced stretches of 10 or 13 consecutive dA residues at different locations throughout the nucleosome. Like that observed with native nucleosomes, they found a poly (dA) tract 40−50 nucleotides from the dyad axis (SHL-4 and -5) stimulates H2A.Z incorporation. These same nucleosomes appear to tighten SWR’s interactions, as reflected by a mild increase in SWR1’s affinity for these nucleosomes.

These data are in remarkable agreement with yeast promoters being AT-rich upstream and downstream of the nucleosome-depleted region and correlate to where H2A.Z is enriched. In this study, the authors find a novel way in which the DNA sequence at gene promoters modulates histone exchange that facilitates transcription. The differences between the current study and that previously reported for SWR is likely due to the inherent difference between strong positioning sequences like the Widom DNA and that of the yeast genome [11,12]. Temperature could also be a factor but alone is not sufficient to completely account for these differences.

Future studies are needed to delve into how the poly (dA) tract stimulates dimer exchange and determines the stage in remodeling that is impacted. It could be that the poly (A) tracts promote “opening” of the nucleosome to facilitate release of the outgoing dimer, which would stimulate dimer exchange. Another possibility is the poly (dA) tract could positively impact the motor domain of SWR and further studies are needed to find if the rate of ATP hydrolysis is changed by this DNA sequence. Also not clear from this study is the subunit and/or domain of the SWR complex whose binding is potentially enhanced by poly (A) at SHL-3 and −4 and how this might stimulate dimer exchange.
